# Case Report: Ischemic central retinal vein occlusion in a patient with relapsed mantle cell lymphoma as a manifestation of suspected cancer-associated hypercoagulability

**DOI:** 10.3389/fmed.2026.1926148

**Published:** 2026-09-15

**Authors:** Chaoxiong Cui, Guanghao Li, Na Liu, Zhenhua Zhang

**Affiliations:** 1 Department of Ophthalmology, Qingdao Central Hospital, University of Health and Rehabilitation Sciences (Qingdao Central Hospital), Qingdao, Shandong, China; 2 Department of General Surgery, Shandong Second Medical University Affilliated Qingdao Eighth People's Hospital - East Campus, Qingdao, Shandong, China

**Keywords:** cancer-associated thrombosis, ischemic central retinal vein occlusion, mantle cell lymphoma, ocular ischemia, thromboembolism

## Abstract

**Background:**

Ischemic central retinal vein occlusion (ischemic CRVO) is a severe, vision-threatening vascular event. While most cases stem from conventional cardiovascular or local anatomical etiologies, its presentation in association with relapsed mantle cell lymphoma (MCL) and suspected cancer-associated hypercoagulability is rare. This report highlights that retinal vascular occlusion in a patient with relapsed MCL represents a rare ocular manifestation of severe systemic prothrombotic activity.

**Case presentation:**

An 89-year-old male with established stage IV MCL (CD20+, cyclin D1+, SOX-11+, Ki-67∼10%), whose systemic relapse had been documented 6 months prior, developed sudden, painless vision loss in his right eye (best-corrected visual acuity, BCVA 0.01) while hospitalized for disease management. Multimodal ophthalmic examination and optical coherence tomography (OCT) supported the diagnosis of ischemic CRVO with prominent macular edema. Laboratory workup revealed marked coagulation abnormalities (D-dimer 4.34 mg/L FEU, Fibrinogen Equivalent Units; antithrombin activity 78%). Cranial MRI (magnetic resonance imaging) revealed multifocal chronic/non-acute ischemic cerebral changes and periventricular leukoaraiosis without acute diffusion restriction. Following a localized peribulbar injection of triamcinolone acetonide (20 mg), right eye BCVA improved to 0.1, with anatomical reduction of retinal edema. Prophylactic panretinal photocoagulation (PRP) was unfeasible due to posterior capsular opacification and severe systemic frailty. Four months later, the patient developed neovascular glaucoma requiring cyclocryotherapy for palliative pain management.

**Conclusions:**

In patients with active relapsed MCL, ischemic CRVO may occur as a focal manifestation of a generalized prothrombotic state. When severe retinal vascular occlusion presents in patients with advanced hematological malignancies, systemic coagulation assessment and neuroimaging evaluation should be considered to identify underlying prothrombotic risks and subclinical cerebrovascular disease, even in the absence of overt focal neurological deficits.

## 1 Introduction

Ischemic central retinal vein occlusion (ischemic CRVO) is a severe, vision-threatening ophthalmic condition characterized by profound retinal hypoperfusion and widespread vascular compromise (1, 2). Pathophysiologically, severe ischemic retinal vascular occlusion typically arises from severe hemodynamic stagnation or extensive microvascular thrombosis (3). While conventional etiologies predominantly involve giant cell arteritis, severe carotid stenosis, and systemic cardiovascular risk factors, retinal vascular occlusion associated with hematological malignancies—particularly mantle cell lymphoma (MCL)—remains exceptionally rare (4, 5). MCL is an aggressive B-cell non-Hodgkin lymphoma characterized by high disease burden and the potential to induce cancer-associated hypercoagulability complications via tumor-derived procoagulants and systemic inflammatory cytokines (5, 6). Here, we present a rare case of ischemic CRVO in an 89-year-old male with relapsed mantle cell lymphoma and preexisting cerebral ischemic changes, discussing the potential multifactorial contributions including cancer-associated hypercoagulability (7).

**Table 1:** Clinical timeline of diagnosis, interventions, and outcomes.

Time Point	Clinical Findings and Disease Status	Diagnostic Evaluations & Interventions
November 2023	Confirmed stage IV classic MCL (CD20+, Cyclin D1+, SOX-11+, Ki-67∼10%) via CT-guided pleural biopsy.	Baseline oncology workup; initiated close clinical monitoring.
December 2023 - March 2024	Incomplete tumor regression on follow-up chest CT.	Administered 4 cycles of CHOP chemotherapy.
March 2024 - June 2024	Stable disease achieved.	Added ripertamab (600 mg) to CHOP for 3 cycles.
July 2025	Systemic disease relapse confirmed by flow cytometry (bone marrow: 40.03% monoclonal B-cells; pleural fluid: 88.71%). High HBV DNA load (1.26 × 10⁷ IU/mL).	Hospitalized for relapse; started entecavir, tenofovir, and dexamethasone tumor-reduction therapy.
October - November 2025	Relapse progression; developed bone marrow suppression and clinical intolerance.	Initiated zanubrutinib (80–160 mg bid); discontinued late Nov. Maintained on methylprednisolone.
December 14, 2025	Painless visual loss OD (BCVA: 0.01 OD, 0.8 OS) following P. aeruginosa bacteremia. Bilateral pseudophakia with moderate PCO.	Fundus photography and OCT were consistent with the diagnosis of ischemic CRVO. Full anticoagulation and intravitreal injections withheld.
December 16–19, 2025	Cranial MRI: multifocal chronic/non-acute ischemic lesions and leukoaraiosis. Coagulation: D-dimer 4.34 mg/L FEU, antithrombin 78%, Padua score 5. Non-stenotic carotid plaques.	Administered right peribulbar injection of triamcinolone acetonide (20 mg) and topical pranoprofen.
December 31, 2025	BCVA OD improved to 0.1 at 2 weeks; OCT showed structural resolution of retinal edema.	Prophylactic PRP deferred due to PCO and critical systemic frailty. Discharged to oncological supportive care.
April 2026	Severe right ocular pain and headache (4 months post-onset); diagnosed with neovascular glaucoma OD (BCVA: NLP, 360° iris rubeosis, IOP T + 3).	Underwent cyclocryotherapy OD (April 7); achieved complete pain relief (IOP Tn∼T + 1 at discharge).
June 2026	Readmitted for MCL progression complicated by opportunistic pulmonary infections. Right eye stable and painless (NLP).	Conservative, comfort-oriented palliative management per family consensus.

MCL, mantle cell lymphoma; CHOP, cyclophosphamide 0.8 g on day 1, epirubicin 70 mg on days 1- 2, vincristine 2 mg on day 1, and prednisone 100 mg on days 1–5; HBV, hepatitis B virus; BCVA, best-corrected visual acuity; OD, right eye; OS, left eye; ischemic CRVO, ischemic central retinal vein occlusion; OCT, optical coherence tomography; PCO, posterior capsular opacification; MRI, magnetic resonance imaging; FEU, fibrinogen equivalent units; PRP, panretinal photocoagulation; NLP, no light perception; IOP, intraocular pressure.

## 2 Case presentation

In November 2023, an 87-year-old male was diagnosed with stage IV classic mantle cell lymphoma (MCL) via a CT-guided pleural biopsy (6). Immunohistochemical analysis demonstrated positive staining for CD20, cyclin D1, and SOX-11, with a Ki-67 proliferation index of approximately 10%. The patient had a 20-year history of hypertension managed with oral felodipine and telmisartan, a 10-year history of coronary artery disease, and a 10-year history of gout, with no history of smoking, prior thromboembolism or diabetes. Initial baseline echocardiography revealed aortic valve calcification with mild regurgitation and mild tricuspid regurgitation, while serial electrocardiograms (ECGs) consistently demonstrated sinus rhythm without evidence of atrial fibrillation. Between December 2023 and March 2024, the patient completed four cycles of CHOP chemotherapy (cyclophosphamide 0.8 g on day 1, epirubicin 70 mg on days 1–2, vincristine 2 mg on day 1, and prednisone 100 mg on days 1–5). Because follow-up CT demonstrated incomplete tumor regression, targeted therapy with ripertamab (anti-CD20 monoclonal antibody, 600 mg) was added to the CHOP regimen for three additional cycles from March to June 2024, achieving stable disease.

The patient remained stable under routine outpatient surveillance until late June 2025, when he developed progressive dyspnea and fatigue. Systemic disease relapse was confirmed in July 2025: whole-body CT demonstrated generalized lymphadenopathy and worsening bilateral pleural effusion; bone marrow aspirate flow cytometry revealed 40.03% CD5+ CD10- monoclonal small B-lymphocytes with Lambda light-chain restriction; and pleural fluid flow cytometry showed 88.71% identical monoclonal B-lymphocytes. High hepatitis B virus (HBV) DNA replication was also identified (1.26 × 10⁷ IU/mL), prompting antiviral therapy with entecavir and tenofovir alongside dexamethasone tumor-reduction therapy. In October 2025, the patient commenced second-line targeted therapy with zanubrutinib (80 mg to 160 mg twice daily, po) (8, 9). In late November 2025, zanubrutinib was discontinued due to poor clinical tolerance and bone marrow suppression; disease management was subsequently maintained with low-dose methylprednisolone (7.5 mg/d tapered to 3 mg/d).

On December 14, 2025, during hospitalization following treatment for a *Pseudomonas aeruginosa* bloodstream infection, the patient experienced sudden, painless vision loss in his right eye (OD). Ophthalmic examination revealed a best-corrected visual acuity (BCVA) of 0.01 in the right eye and 0.8 in the left eye. Bilateral intraocular pressure (IOP) measured 15 mmHg. Anterior segment examination showed transparent corneas, quiet anterior chambers, and pseudophakic eyes bilaterally with moderate posterior capsular opacification (PCO). The right pupil exhibited a sluggish light reflex compared to the left, consistent with a relative afferent pupillary defect (RAPD). Fundus photography of the right eye demonstrated extensive flame-shaped and blot retinal hemorrhages across all four quadrants, diffuse vascular tortuosity and venous dilatation, and focal arterial attenuation without a cherry-red spot (Figure 1A). Horizontal optical coherence tomography (OCT) B-scans revealed prominent inner retinal hyper-reflectivity and pronounced structural thickening secondary to severe retinal ischemia, accompanied by marked subretinal fluid (SRF) and intraretinal cystoid spaces reflecting hydrostatic venous transudation (Figure 1C). Together, these ophthalmic and tomographic findings were consistent with the diagnosis of ischemic CRVO. Fundus photography and OCT of the left eye demonstrated features consistent with early non-exudative age-related macular degeneration, characterized by mild pigmentary alterations and focal sub-RPE (retinal pigment epithelium) drusenoid elevations without associated intraretinal or subretinal fluid (Figure 1B, D). Fundus fluorescein angiography (FFA) and optical coherence tomography angiography (OCTA) were deferred due to advanced age, systemic frailty, and acute illness.

**Figure 1:**
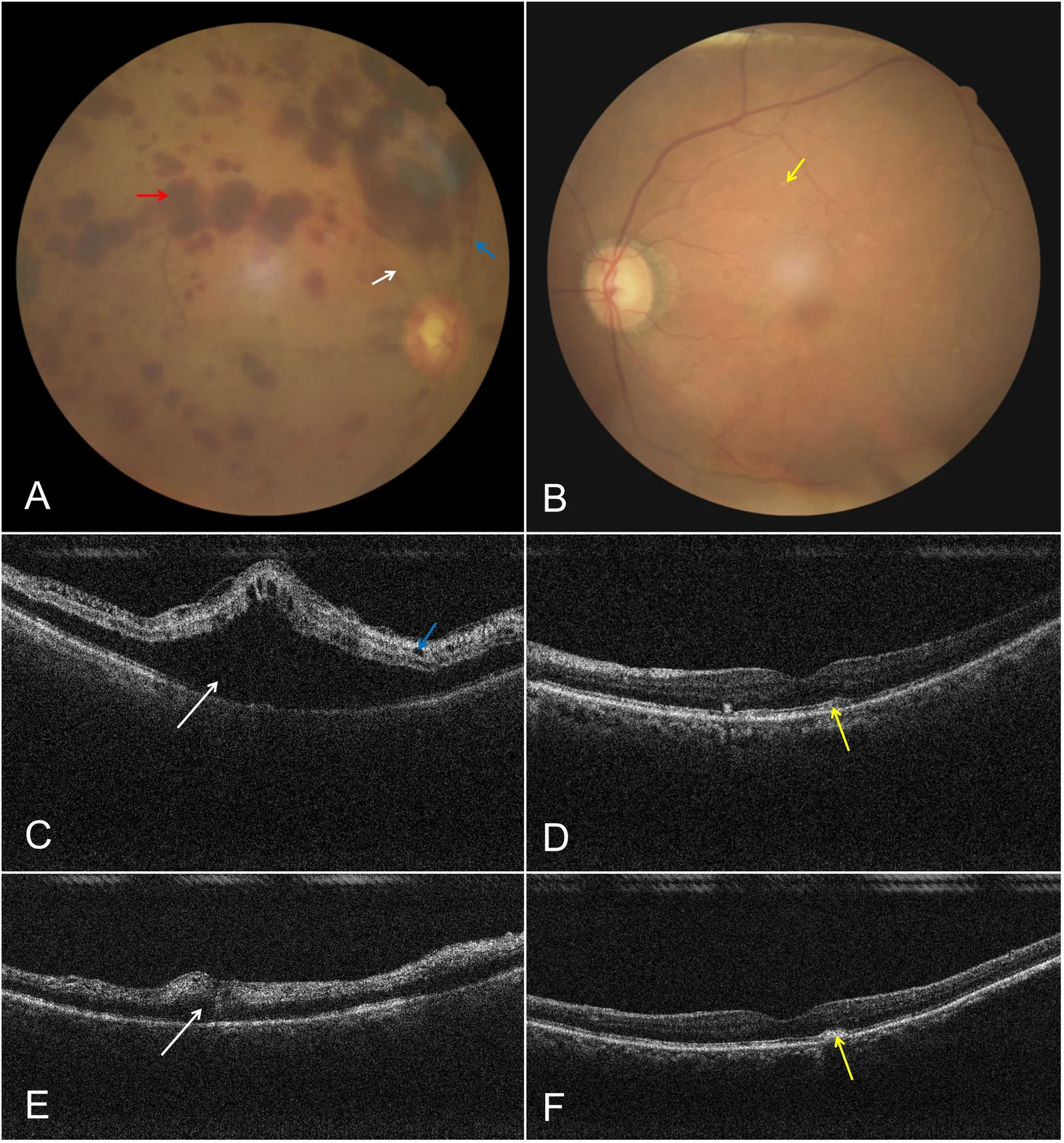
Bilateral multimodal retinal imaging at baseline and follow-up. **(A)** Color fundus photograph of the right eye at presentation demonstrates extensive flame-shaped and blot retinal hemorrhages (red arrow), diffuse vascular tortuosity and venous dilatation (blue arrow), and focal arterial attenuation (white arrow) without a cherry-red spot. **(B)** Color fundus photograph of the unaffected left eye at presentation shows mild dry age-related macular changes, including faint drusen (yellow arrows). **(C)** Baseline horizontal OCT B-scan of the right eye reveals prominent inner retinal hyper-reflectivity and structural thickening secondary to severe retinal ischemia, accompanied by prominent subretinal fluid (white arrow) and intraretinal cystoid spaces (blue arrow). **(D)** Baseline horizontal OCT B-scan of the left eye reveals preserved foveal contour with focal, dome-shaped sub-RPE drusenoid elevations (yellow arrows) and no retinal fluid. **(E)** Follow-up OCT of the right eye two weeks post-treatment demonstrates anatomical improvement with substantial reabsorption of subretinal fluid (white arrow) and reduction of retinal thickening. **(F)** Follow-up OCT of the left eye over the same interval confirms stable macular architecture with unchanged sub-RPE elevations (yellow arrow) and no interval exudation.

To assess potential systemic thromboembolic involvement, cranial and orbital MRI and carotid duplex ultrasonography were performed on December 16, 2025. Orbital MRI showed unremarkable bilateral orbits and optic nerves without mass lesions or lymphomatous infiltration. Cranial MRI revealed multifocal ischemic lesions involving the bilateral basal ganglia, corona radiata, and bilateral frontal, temporal, and parietal subcortical white matter, characterized by punctate and patchy hyperintensities on T2-weighted and FLAIR (fluid-attenuated inversion recovery) sequences without acute diffusion restriction on DWI (diffusion-weighted imaging) (Figure 2). Confluent periventricular hyperintensities were noted bilaterally, consistent with chronic white matter changes, along with mild ventricular enlargement and sulcal prominence indicative of age-related cerebral involution. The cerebellar hemispheres, pituitary gland, and midline structures appeared unremarkable. Carotid ultrasonography demonstrated bilateral intimal-medial thickening with plaque formation but no hemodynamically significant stenosis or vessel wall inflammatory edema. Coagulation profiling on December 19, 2025, revealed profound hypercoagulability: plasma D-dimer was elevated at 4.34 mg/L FEU (normal <0.5 mg/L), antithrombin activity was reduced to 78% (normal 80%–120%), PT was 14.6 s, aPTT was 30.1 s, and fibrinogen was 2.34 g/L (10, 11). The Padua prediction score was calculated as 5 points, indicating high venous thromboembolism (VTE) risk (10). Giant cell arteritis (GCA) was considered unlikely given the absence of localized temporal headache, scalp tenderness, jaw claudication, or abnormal temporal artery palpation, alongside unremarkable carotid and cranial imaging.

**Figure 2:**
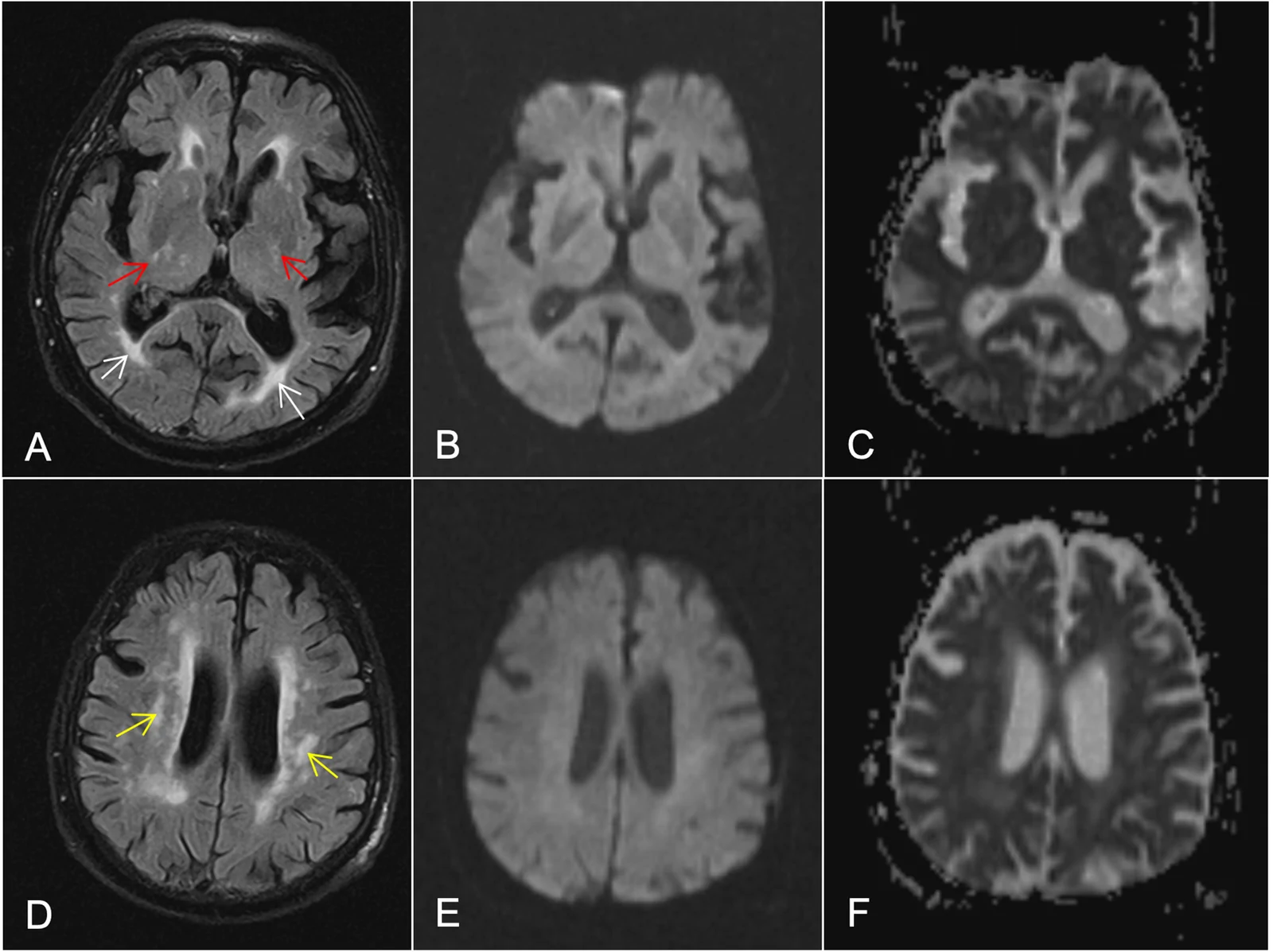
Cranial MRI findings. Axial FLAIR images **(A,D)**, DWI **(B,E)**, and ADC maps **(C,F)** at the basal ganglia **(A–C)** and corona radiata **(D–F)** levels. **(A,D)** Axial FLAIR shows hyperintense ischemic foci in the bilateral basal ganglia (red arrows) and bilateral corona radiata (yellow arrows), alongside periventricular white matter hyperintensities (white arrows). **(B,C,E,F)** Corresponding DWI and ADC maps show no acute diffusion restriction, ruling out acute cerebral infarction and confirming chronic ischemic changes.

Following multidisciplinary consultation among ophthalmology, hematology, and neurology, therapeutic management was established. Full systemic anticoagulation was withheld due to a high risk of major hemorrhage associated with advanced age (89 years old) and extensive active intraretinal hemorrhages (11). Intravitreal anti-VEGF (vascular endothelial growth factor) or corticosteroid injections were avoided due to the heightened risk of endophthalmitis in the setting of recent bloodstream infection and immunocompromised status. To achieve rapid bedside reduction of macular edema without intraocular entry, a localized peribulbar injection of triamcinolone acetonide (TA; 20 mg/0.5 mL, Tongxitong, Kunming Jida Pharmaceutical Co., Ltd., Yunnan, China; 40 mg/1 mL) was administered to the right eye on December 17, 2025, combined with topical pranoprofen eye drops four times daily. Under sterile conditions, 0.1 mL of 0.2% lidocaine was infiltrated at the skin entry site. A 27-gauge, 35-mm needle was introduced at the junction of the middle and lateral thirds of the inferior orbital rim to a depth of 20–25 mm. Following negative aspiration for blood, the suspension was slowly injected into the peribulbar space. Firm digital pressure with sterile gauze was maintained over the puncture site for 10 min immediately after needle withdrawal. The patient was monitored for 30 min, during which vital signs, intraocular pressure, and gross vision remained stable without evidence of hematoma formation. The peribulbar injection was well tolerated without elevation of intraocular pressure or local complications.

At the two-week follow-up (December 31, 2025), right eye BCVA improved to 0.1. Horizontal OCT demonstrated anatomical regression with substantial reduction in inner retinal thickening and reabsorption of SRF (Figure 1E). Prophylactic panretinal photocoagulation (PRP) was not performed during this admission because optical media opacity from posterior capsular opacification impaired clear retinal delivery, and the patient's critical physical debility precluded prolonged sitting at a slit lamp. Following discharge, the patient's subsequent clinical care was entirely centered on bedridden supportive oncological management, resulting in a hiatus in scheduled outpatient ophthalmology visits.

In April 2026 (four months post-onset), the patient presented with severe right ocular pain and headache. Examination revealed neovascular glaucoma (NVG) OD with loss of light perception (BCVA: NLP, no light perception), 360-degree prominent iris rubeosis, and elevated IOP (digital palpation T + 3) (Figure 3). On April 7, 2026, cyclocryotherapy was performed under local anesthesia. The procedure successfully relieved ocular pain, and at discharge, digital intraocular pressure was controlled within the normal to mildly elevated range (Tn∼T + 1). In June 2026, the patient was readmitted for lymphoma progression complicated by opportunistic pulmonary infections. Palliative comfort care was prioritized per family consensus, and the patient remained stable under supportive management.

**Figure 3:**
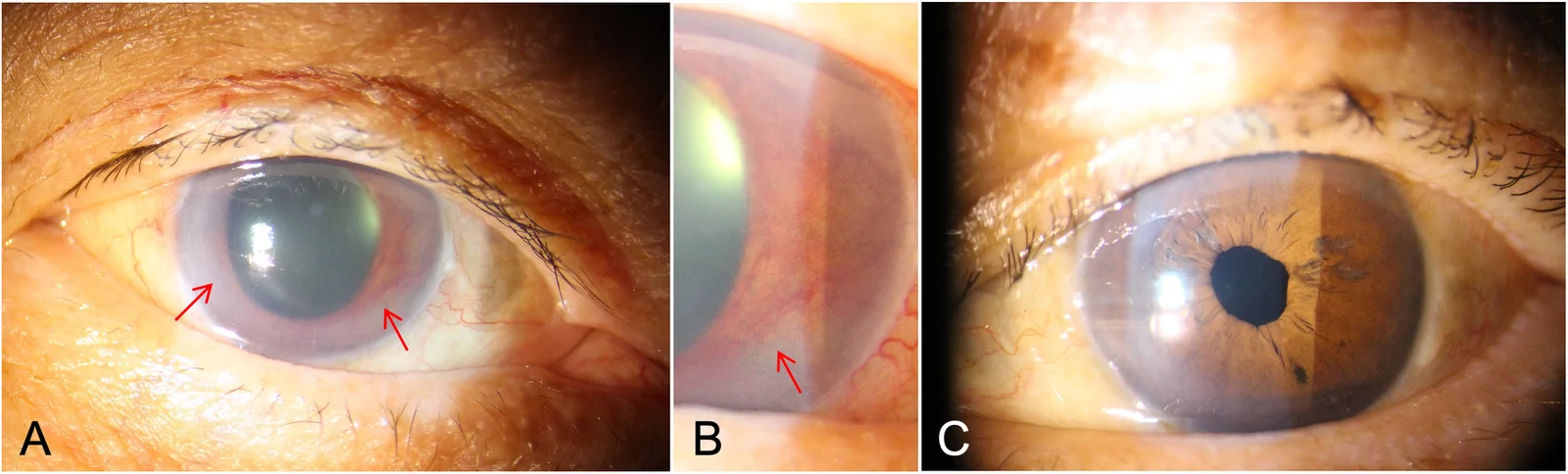
Slit-lamp anterior segment photographs. **(A)** Slit-lamp photograph of the right eye shows 360-degree iris rubeosis (red arrows) and a dilated pupil through which an intraocular lens is faintly visible secondary to corneal edema. **(B)** Magnified view of the iris stroma shows fine, branching neovascular vessels (red arrow). **(C)** Slit-lamp photograph of the fellow left eye shows a quiet anterior segment, clear cornea, and normal iris architecture with an *in situ* intraocular lens.

## 3 Discussion and conclusions

Ischemic CRVO in the setting of relapsed mantle cell lymphoma (MCL) is an exceptionally rare vascular presentation associated with a multifactorial prothrombotic state (1, 5). Severe retinal venous outflow obstruction and profound microvascular ischemia typically indicate either intense systemic hypercoagulability or local vascular compromise within the optic nerve sheath. In this patient, the temporal presentation of ischemic retinal vascular occlusion alongside markedly elevated D-dimer (4.34 mg/L FEU), decreased antithrombin activity, and a background of chronic ischemic cerebral lesions suggests a suspected multifactorial prothrombotic state in which active hematologic malignancy may have played a contributory role (7, 10).

Cancer-associated hypercoagulability in hematological malignancies arises from complex interactions involving tumor-derived tissue factor expression, inflammatory cytokine release, platelet hyperreactivity, and accelerated thrombin generation (5, 10, 11). Conventional vascular risk factors, including advanced age, hypertension, and carotid atherosclerotic plaques—likely contributed to baseline vascular vulnerability. Against this background, the emergence of ocular vascular occlusion in a patient with active MCL supports a multifactorial etiology, where cancer-associated hypercoagulability may have acted as a compounding trigger alongside underlying cardiovascular disease (10). Furthermore, although targeted agents such as BTK (Bruton Tyrosine Kinase) inhibitors (zanubrutinib) carry known vascular toxicities, zanubrutinib had been discontinued three weeks prior to the ocular event due to bleeding tendencies rather than thrombosis, suggesting that acute drug toxicity was unlikely the primary trigger (12).

Managing severe retinal vascular occlusion in elderly patients with advanced malignancies presents substantial therapeutic challenges. Systemic anticoagulation represents standard therapy for cancer-associated thrombosis (10, 11). However, it is frequently contraindicated in patients with active bleeding risks (11). In this patient, full systemic antithrombotic therapy was withheld due to extensive active intraretinal hemorrhages (11). Localized peribulbar triamcinolone acetonide was selected as a pragmatic bedside periocular corticosteroid approach to alleviate macular edema while eliminating intraocular injection risks in a frail patient recovering from bacteremia (13).

Although functional vision improved modestly from 0.01 to 0.1 at two weeks alongside anatomical reduction of macular edema, this clinical change cannot be definitively attributed to the peribulbar triamcinolone injection alone. In severe ischemic CRVO, macular edema and intraretinal fluid may fluctuate over the natural disease course, and transient structural improvement does not halt the underlying ischemic trajectory (14). Extensive retinal non-perfusion inevitably drove subsequent anterior segment neovascularization. Prophylactic panretinal photocoagulation (PRP) was not feasible during the early convalescent stage because posterior capsular opacification impaired retinal visualization, while the patient's critical systemic debility precluded prolonged sitting at a slit lamp. Following discharge, clinical care centered entirely on bedridden oncological support, preventing outpatient retinal ablation before neovascular glaucoma developed at four months. Palliative cyclocryotherapy served as an effective globe-preserving intervention to achieve durable pain relief and intraocular pressure stabilization in this late stage.

From the patient perspective, although advanced age (89 years old), critical illness, and disease progression precluded a formal first-person written narrative, the patient and family expressed meaningful satisfaction with the short-term restoration of ambulatory vision (BCVA 0.1) following the peribulbar injection, which aided orientation and daily communication during acute hospitalization. In the later terminal stage, the family appreciated the prompt pain relief achieved through cyclocryotherapy and fully supported conservative, comfort-oriented palliative care.

This report has several strengths, including longitudinal clinical documentation from initial MCL diagnosis through relapse, multimodal structural and neuroimaging validation, and multidisciplinary coordination across ophthalmology, hematology, and neurology. Nevertheless, important limitations must be acknowledged: first, as a single observational case, direct pathological causality between cancer-associated hypercoagulability and retinal vascular occlusion cannot be definitively established (7); second, invasive evaluations including fundus fluorescein angiography (FFA) and OCT angiography (OCTA) were deferred due to critical systemic frailty; third, quantitative central retinal thickness mapping could not be retrospectively extracted because only rapid single-line B-scans were acquired instead of dense volumetric scans due to poor patient fixation and fatigue, limiting our OCT analysis to qualitative structural assessment; fourth, confounding contributions from underlying vascular disease (hypertension, carotid plaques) cannot be entirely excluded; and fifth, systemic anticoagulation could not be safely administered to assess therapeutic responsiveness (11).

In conclusion, ischemic CRVO in patients with active hematological malignancies should raise awareness of potential systemic hypercoagulability and coexisting subclinical cerebrovascular disease. Systemic coagulation screening and neuroimaging evaluation may be considered in patients with active hematological malignancies presenting with severe retinal vascular occlusion to characterize underlying vascular risk profiles, even in the absence of overt focal neurological deficits (7). For frail or elderly patients with limited therapeutic options, individualized localized interventions can achieve palliative visual optimization and subsequent comfort control throughout the disease trajectory.

## Data Availability

The original contributions presented in the study are included in the article/Supplementary Material, further inquiries can be directed to the corresponding author.
